# Adolescent Idiopathic Scoliosis Minimum Data Set: Towards Standardization of Data Elements in History and Physical Examination

**DOI:** 10.7759/cureus.58332

**Published:** 2024-04-15

**Authors:** Jenny L Zheng, Ying Li, Grant Hogue, Megan Johnson, Jason B Anari, Keith D Baldwin

**Affiliations:** 1 Orthopedic Surgery, Children's Hospital of Philadelphia, Philadelphia, USA; 2 Orthopedic Surgery, C.S. Mott Children's Hospital, Ann Arbor, USA; 3 Orthopedic Surgery, Boston Children’s Hospital, Boston, USA; 4 Orthopedic Surgery, Scottish Rite for Children, Dallas, USA; 5 Division of Orthopedics, Children's Hospital of Philadelphia, Philadelphia, USA

**Keywords:** minimum data set, physical exam, history, nonoperative care, adolescent idiopathic scoliosis

## Abstract

Introduction: Nonoperative care represents a cornerstone of adolescent idiopathic scoliosis (AIS) management, although no consensus exists for a minimal data set. We aimed to determine a consensus in critical data points to obtain during clinical AIS visits.

Methods: A REDCap-based survey was distributed to Pediatric Orthopedic Society of America (POSNA), Pediatric Spine Study Group (PSSG), and International Society on Scoliosis Orthopedic and Rehabilitation Treatment (SOSORT). Respondents ranked the importance of data points in history, physical examination, and bracing during AIS visits.

Results: One hundred eighty-one responses were received (26% response rate), of which 86% were physicians and 14% were allied health professionals. About 80% of respondents worked at pediatric hospitals or pediatric spaces within adult hospitals, and 82% were academic, with the majority (57%) seeing 150+ unique AIS patients annually. Most respondents recommended six-month follow-up for patients under observation (60%) and bracing (54%). Most respondents (75%) considered family history and pain important (69%), with the majority (69%) asking about pain at every visit. Across all time points, Adam’s forward bend test, shoulder level, sagittal contour, trunk shift, and curve stiffness were all considered critically important (>60%). At the first visit, scapular prominence, leg lengths, motor and neurological examination, gait, and iliac crest height were also viewed as critical. At the preoperative visit, motor strength and scapular prominence should also be documented. About 39% of respondents use heat sensors to monitor bracing compliance, and average brace wear since the prior visit was considered the most important (85%) compliance data point.

Conclusions: This study establishes recommendations for a 19-item minimum data set for clinical AIS evaluation, including history, physical exam, and bracing, to allow for future multicenter registry-based studies.

## Introduction

Adolescent idiopathic scoliosis (AIS) is one of the most common disorders treated by pediatric orthopedic surgeons, with a global prevalence of up to 5.2% [1-4]. Nonoperative office care represents a cornerstone of AIS care and is the only orthopedic treatment required by a vast majority of patients with AIS [1,3,5]. Although significant progress has been made in the areas of nonoperative care and bracing, there is still great heterogeneity among clinical practices [6,7]. A systematic review completed by the United States Preventive Services Taskforce (USPSTF) on scoliosis screening was unable to perform meta-analyses of results for scoliosis screening due to the heterogeneity of study results [8]. Large-scale registry studies are necessary to elucidate optimal nonoperative care, although a lack of consensus regarding common data elements of AIS office visits has rendered such studies difficult. Subsequently, there is a marked need to establish common data elements for study in the field of non-operative care of AIS as previously produced by organizations for other common pathologies such as sports-related concussions and spinal cord injuries [9,10].

The purpose of this study was to determine a majority consensus of important data elements during clinical AIS visits through a cross-sectional survey of the membership of multiple large organizations of physicians and allied health professionals who treat scoliosis.

## Materials and methods

Survey development

A questionnaire was developed across four institutions involved in the Scoliosis Non-Operative REgistry Study (SNORES) Group, a collaboration developed with funding from the Scoliosis Research Society and dedicated to the study and improvement of non-operative treatment of adolescent idiopathic scoliosis. The questionnaire examined various aspects of clinical AIS visits, including patient histories, physical examination measurements, radiographic examination practices, and bracing at a patient’s first visits, subsequent visits, immediate preoperative visits, and final/brace wean visits. Survey questions asked respondents to either rate the importance of data elements as very to not important or rank elements from most to least important.

The survey structure was divided into the following sections: (1) respondent demographics, (2) patient history, (3) physical examination, (4) radiographic examination, and (5) other elements of visits, including follow-up frequency, MRI recommendations, and bracing practices. Adaptive branching questions were utilized to better elucidate provider preferences. An optional text field was included at the end of the survey for respondents to recommend additional data elements that were not included in the survey. The longest version of the survey contained 132 total fields and required less than 10 minutes to complete.

Survey beta-testing was performed by two attending pediatric orthopedic surgeons and four pediatric orthopedic surgery clinical fellows from a single institution. Survey question wording was improved based on feedback from the beta testers. Institutional Review Board (IRB) exemption was obtained from our local institution (IRB 22-019843, Protocol Title: SNORES Survey Studies).

Survey distribution

The questionnaire was distributed via REDCap to 1400 members of the Pediatric Orthopedic Society of America (POSNA), 180 members of the Pediatric Spine Study Group (PSSG), and 224 members of the International Society on Scoliosis Orthopedic and Rehabilitation Treatment (SOSORT). The survey was reviewed and approved for distribution to the POSNA membership by the POSNA Evidence-Based Practice Committee, to the PSSG membership by the PSSG Executive Director, and the SOSORT membership by the SOSORT Scientific Committee.

POSNA, PSSG, and SOSORT members were invited via email to participate in the survey. The survey link was also shared on Twitter by members of the research team. A short description of the survey was provided prior to the opt-in questions. Respondents could opt-in if they had experience managing AIS, were willing to voluntarily participate, and had not previously completed the survey. Informed consent was obtained by successfully answering the opt-in questions. Anonymous responses were collected over a five-month period.

Analysis

Survey responses with at least the first three sections completed were included in our final analysis. Results from the survey portions surrounding clinical evaluation and care were analyzed. Standard descriptive statistics were used to evaluate survey responses, including frequencies of answers. Based on the number of POSNA members who self-identified as spine specialists (280), the total number of PSSG members (180), and the total number of SOSORT members (224) at the time of survey distribution, we estimated an overall response rate of 26%.

The perceived importance of data elements was stratified into quartiles by the aggregate number of respondents who viewed each element as either important or very important. Recommendations for a minimum data set were established based on majority agreement on the importance of evaluated data elements. Physical examination elements were defined as critical if a supermajority of 60% of respondents perceived them as important. The 60% threshold, based on a binominal distribution of all evaluated responses, provides a 99% confidence interval that the normal bound of favorable responses is greater than 50%.

## Results

Responses and demographics

A total of 181 responses (26% response rate) were reviewed from the United States and 22 other countries (Table 1). An additional 79 survey responses were received but excluded from the analysis due to a lack of completion (68% completion rate). Of the respondents, 86% were physicians and 14% were allied health professionals. 80% of respondents worked at a free-standing pediatric hospital or in a pediatric space within an adult hospital, and 82% were academic, with the majority (57%) seeing 150+ unique AIS patients per year. There were no significant qualitative differences in results on subgroup analyses by provider types and characteristics. Most respondents recommended six-month follow-up intervals for patients under observation (60%) or undergoing bracing (54%).

**Table 1:** Respondent demographics.

Demographics	N=181	%
Role		
Physician	156	86.2
Physical therapist	16	8.8
Orthotist	6	3.3
Chiropractor	3	1.7
Type of hospital		
Free-standing pediatric hospital	111	61.3
Pediatric practice within space inside adult hospital	34	18.8
Community/general hospital	10	5.5
Other	26	14.4
Type of practice		
Academic	149	82.3
Non-academic	30	16.6
Years in practice		
0-10	57	31.5
11-20	52	28.7
21-30	32	17.7
30+	37	20.4
AIS patients seen annually		
0-25	7	3.9
26-50	17	9.4
51-100	31	17.1
101-150	23	12.7
151+	103	56.9
Geographic location		
United States	129	71.3
Northeast	33	18.2
Midwest	20	11.0
West	23	12.7
South	30	16.6
Puerto Rico	2	1.1
Canada	6	3.3
Europe	27	14.9
Asia	8	4.4
South America	3	1.7
Australia	3	1.7

Patient history

**Figure 1:**
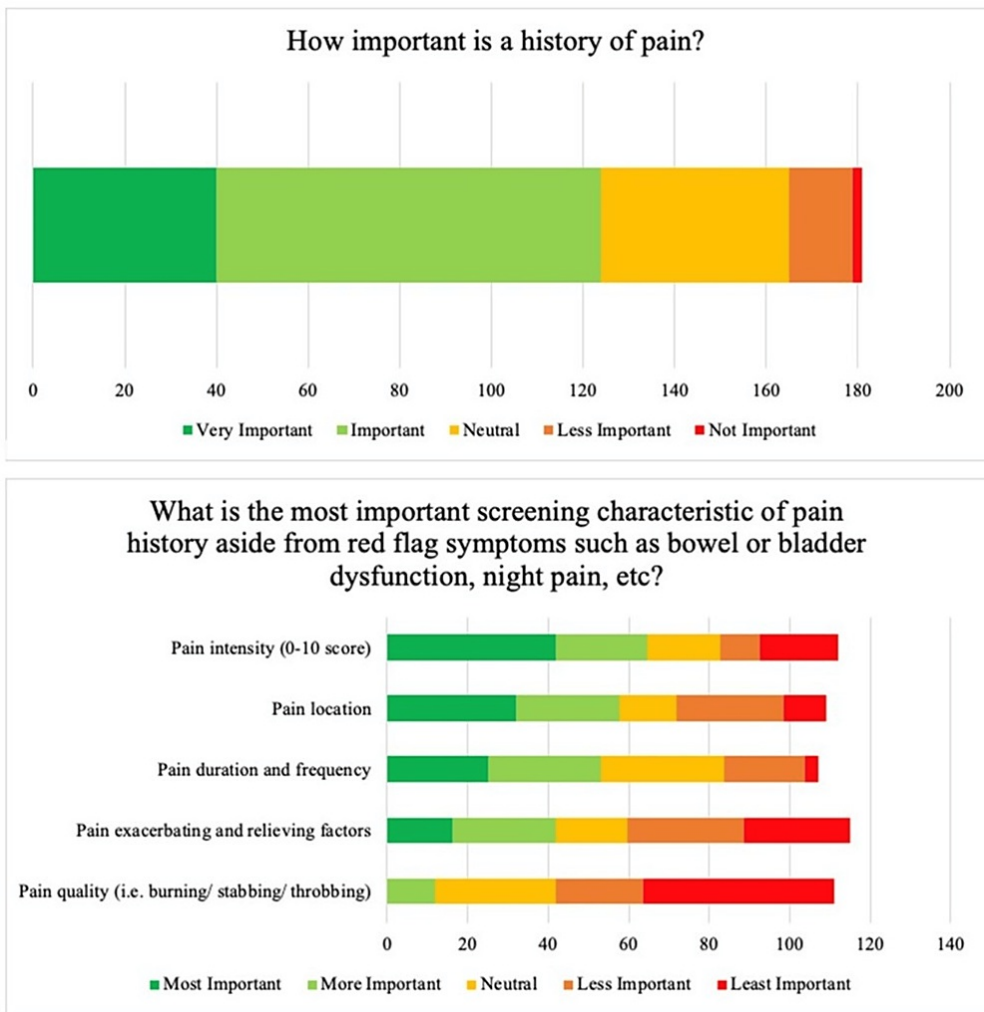
Respondent preferences on obtaining family histories.

**Figure 2:**
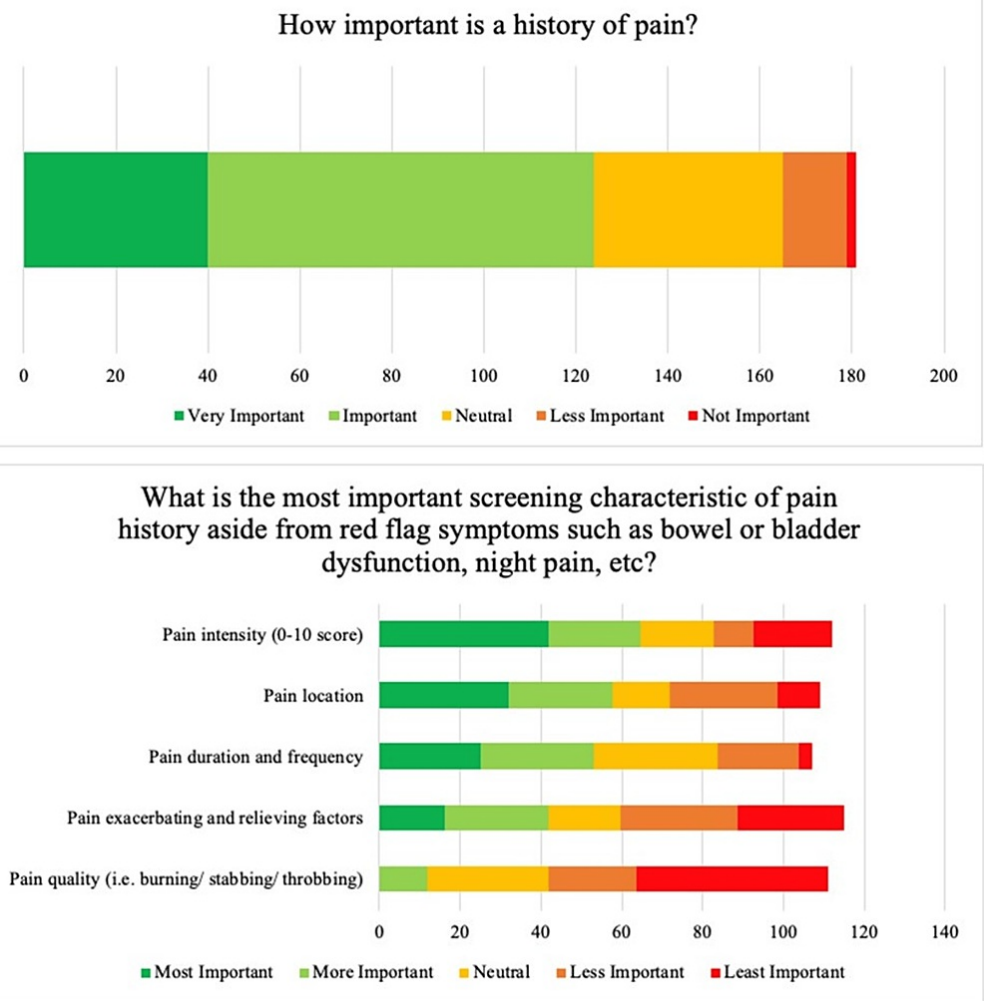
Respondent preferences on obtaining histories to evaluate for pain.

Most respondents (75%) considered family history important, although with variability regarding specific factors of family history (Figure 1). Within this cohort, respondents considered the degree of relatives with scoliosis (e.g., mother vs aunt) to be most important (47%), followed by surgeries received by those relatives (40%), number of relatives with scoliosis (35%), magnitude of the relative’s scoliosis (30%), and lastly, other associated congenital/neurological history (30%). Respondents also considered pain important (69%), with the majority (69%) asking about pain at every visit (Figure 2). Pain intensity was perceived to be the most important characteristic when evaluating pain (52%), followed by location (47%), then duration/frequency (43%), exacerbating/relieving factors (34%), and finally, quality (10%). Menarche status was considered important by over 60% of respondents at the first visit and follow-up visits.

Physical examination

**Figure 3:**
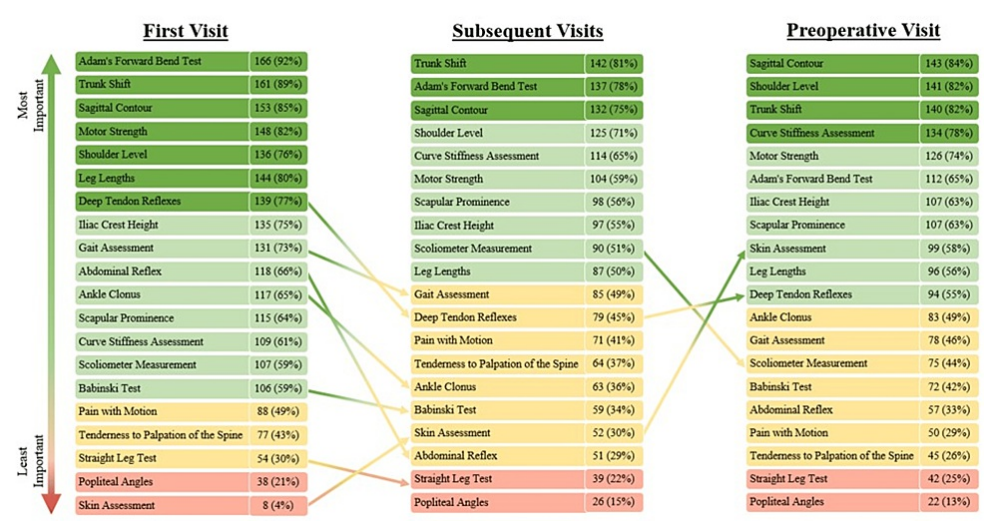
Respondent ranking of the importance of physical examination data points at each visit type. Colors represent each quartile of importance, stratified by the aggregate number of respondents who ranked the data element as important or very important.

The ranked list of the importance of physical exam elements is shown in Figure 3 and grouped into quartiles by the proportion of respondents who perceived each element as important. From the first visit to subsequent follow-up visits, the proportion of respondents who viewed skin assessment as important or very important increased (4% to 30%), while the proportion of respondents who viewed deep tendon reflexes (77% to 45%), gait (73% to 49%), abdominal reflexes (66% to 29%), ankle clonus (65% to 36%), and Babinski test (59% to 34%) as important decreased. At the preoperative visit, more respondents viewed skin assessment (30% to 58%) and deep tendon reflexes (45% to 55%) as important compared to previous visits. The least important physical exam findings across all time points included popliteal angles, straight leg tests, pain with motion, and tenderness to palpation. Table 2 presents a recommended minimum data set for the history and physical exam, based on these survey results.

**Table 2:** AIS clinical visit minimum data set recommendations. AIS: adolescent idiopathic scoliosis.

Baseline characteristics	First visit	Subsequent visits	Preoperative visit
History			
Do you have immediate family members with a history of scoliosis? (Y/N)	X		
Menarche	X	X	
Do you have pain? (Y/N), pain score (0-10), location of pain	X	X	X
Physical exam			
Adam’s forward bend test	X	X	X
Trunk shift	X	X	X
Sagittal contour	X	X	X
Shoulder level	X	X	X
Curve stiffness assessment	X	X	X
Motor strength	X		X
Scapular prominence	X		X
Leg lengths	X		
Deep tendon reflexes	X		
Abdominal reflexes	X		
Ankle clonus	X		
Gait assessment	X		
Iliac crest height	X		X
Bracing			
Average hours per day in a brace since the last visit (if applicable)		X	X

Bracing

To monitor bracing compliance, 39% of respondents use heat sensors/buttons, while the remaining 61% of respondents use patient-reported compliance. Average brace wear since the prior visit was the most important (85%) compliance data point, followed by average brace wear over the prior month (63%).

## Discussion

Previous studies have shown adolescent idiopathic scoliosis to affect up to 5.2% of the at-risk population, and the vast majority of these children will only require non-operative office care for their spinal deformity [1-5]. However, there is no consensus regarding the most important minimum data set to obtain during clinical AIS visits. Establishing common data elements in the nonoperative care of AIS is critical for decreasing the heterogeneity of information that has hampered the understanding of the natural history and personality of individual curves. This understanding can only come from well-designed large registry studies. This study aimed to elucidate an expert consensus for the evaluation and treatment of AIS among members of POSNA, PSSG, and SOSORT in order to define critical aspects of clinical visits, including various factors in patient histories, physical examinations, and bracing monitoring during a patient’s first visit, follow-up visits, and immediate preoperative visit.

Here, we propose a 19-item minimum data set for clinical AIS visits as a foundational tool for future large registry studies (Table 2). This minimum data set includes brief history questions, physical exam data points, average brace wear since the patient’s last visit, and follow-up interval recommendations by curve magnitude. Our goal was to establish a template that broadly encompasses the majority of AIS patients. However, physicians need to exercise their clinical judgment when managing individual patients with unique presentations and concerns. Although the importance of many of the data elements presented in this study is generally agreed upon, our study sheds light on the large variability of beliefs that persist within the pediatric orthopedic community and renders a large registry study of non-operative AIS care difficult. This study aimed to wrangle variations in clinical AIS care and serves as a call to establish a standardized minimum dataset.

Patient history

When obtaining a patient history, we recommend at-minimum that physicians ask about family history, including whether a first-degree relative received treatment for scoliosis. Of the survey respondents who viewed family history as important, the degree of relatives with scoliosis was considered the most important characteristic by 47% of respondents. This recommendation is supported by a study from Grauers et al. of patients with adolescent and juvenile idiopathic scoliosis, which reported 36% of patients to have at least one first-degree relative with scoliosis and 51% of patients to have one or more relatives with scoliosis [11]. Similarly, Watanabe et al. report a 1.5 times higher odds ratio for AIS for patients whose mothers had scoliosis [12].

Additionally, pain should be evaluated at every visit, including intensity on a scale of 0-10 and location of the pain. The majority of survey participants report obtaining pain histories routinely at every visit and identified pain intensity and location as the most important characteristics when evaluating pain. This recommendation is supported by existing studies that suggest nearly ¼ of AIS patients report back pain at the time of diagnosis and an additional 9% develop back pain during follow-up visits [13]. The odds of back pain are reportedly over two times greater in adolescents with scoliosis compared to those without [14]. As such, we recommend assessing for pain at each visit.

If applicable, menarche status should be assessed at a patient’s first visit and follow-up visits. An estimated 75% of curves from 20 to 30 degrees at the onset of puberty go on to require surgical intervention [15]. Nearly 100% of patients with curves over 30 degrees at the onset of puberty will require surgery [15]. As such, it is important to closely monitor a patient’s menarche status and growth velocity. Skeletal maturity may further be assessed on a radiographic examination.

Physical exam

Adam’s forward bend test, shoulder level, sagittal contour, trunk shift, and curve stiffness should be assessed at every clinical visit to characterize the overall severity of the spinal deformity. Adam’s forward bend test is widely recognized as the best noninvasive clinical test for scoliosis, having high sensitivity (85-100% for thoracic curves) without sacrificing specificity (47-74% for thoracic curves) for identification of axial and coronal plane curvatures [16]. Sagittal contour should also be evaluated, as scoliosis is a three-dimensional deformity and treatment involves deformity in all three planes. Current literature demonstrates a limited understanding of the relationship between coronal and sagittal plane deformities, and routine documentation of sagittal deformities will address this knowledge gap [17,18]. At a minimum, we recommend evaluation of whether the sagittal contour is normal or abnormal, with particular attention to abnormal presentations to hyper- and hypokyphosis. Similarly, the presence or absence of any shoulder imbalance is an important aspect of both treatment methodology and the overall cosmetic appearance of AIS, although the relationship between shoulder asymmetry and overall deformity remains poorly understood [19]. Previous studies have also emphasized the high prevalence of trunk imbalance in AIS and the importance of repeated screening as patients mature [20]. Documentation of trunk shift will also help future studies better identify factors to predict the progression of trunk imbalance [21]. Finally, curve flexibility is used as an important aspect of level and therefore a critical factor, particularly when considering the surgical management of AIS [22]. At minimum, clinicians should note whether curves are flexible or rigid, although more objective measurements of curve flexibility may be better assessed on radiographic examination.

At a patient’s first office visit, based on the results of our survey, we recommend physicians also assess for scapular prominence, leg lengths, motor strength, deep tendon reflexes, abdominal reflexes, ankle clonus, gait, and iliac crest height. Scapular asymmetry is often notable in patients with AIS. Leg lengths should be assessed to identify potential leg length discrepancies that may result in functional scoliotic curves. Furthermore, patients with AIS do not typically present with neurological deficits. Particular attention should be paid to motor strength, deep tendon reflexes, abdominal reflexes, and ankle clonus, in addition to red-flag symptoms such as bowel or bladder incontinence. Advanced imaging should be considered in the presence of neurological deficits [23]. The presence of antalgic gait patterns and iliac crest height should also be evaluated, as several previous studies have shown pelvic rotation and alteration of iliac spine geometry to be more severe in patients with more severe AIS, and further research is needed to understand the relationship between pelvic morphological changes and increasing scoliotic curve magnitude [24].

Preoperatively, in addition to the physical exam elements recommended across all visits, motor strength and scapular prominence should be documented. Overall, physicians must ensure adequate exposure of the back and extremities if needed to properly assess all of the above data elements.

Bracing

For patients undergoing bracing, clinicians should at least record the average number of hours the brace has been worn per day since the patient’s last visit. This metric was viewed as important by 87% of our survey respondents. Interestingly, 40% of survey participants reported having access to compliance buttons/sensors. Previous studies have shown that patient histories overestimate time in brace [25]. As such, we recommend the use of objective heat-sensor compliance monitoring when available rather than patient-provided histories. Increased use of monitoring to collect objective brace wear compliance data will allow for large-scale registries to determine the minimum effective brace wear time needed to lower a patient’s risk of progression into the surgical range [26].

Follow-up

We recommend orthopedic follow-up every six months for patients undergoing bracing and observation. Our survey showed that six-month follow-up intervals were preferred by 54% of respondents for bracing patients and by 60% of respondents for patients under observation. These intervals are consistent with recommendations in the current literature [4,20-28]. Twelve-month intervals may be considered for patients under observation with a low risk of progression [28]. 

Limitations

This study had several limitations inherent to the nature of the survey methodology. In order to reduced survey length, redundancy, and respondent fatigue, we were unable to include every element of clinical AIS evaluation and care in survey response options. In particular, this study was unable to correlate visit time points in association with different stages of skeletal maturity. The goal of this study was to establish a template for clinical AIS care that encompasses the majority of patients. Physicians should continue to exercise their own clinical judgment when evaluating patients at varying degrees of skeletal maturity and their unique presentations. Additionally, while our survey was distributed to the entire membership of POSNA, PSSG, and SOSORT, the overall response rate was estimated to be just over 1/4 of the relevant members.

While survey studies appear to have grown in popularity, response rates appear to be declining, possibly due to respondent fatigue. A systematic review of POSNA survey studies from 1991 to 2017 identified a mean response rate of 42% ± 19% and a downward trend over the study period [29]. Other studies have identified response rates as low as 15% among orthopedic surgeons [30]. While our study’s sample size captures a wide geographic and demographic distribution, our results may not represent the heterogeneity of practices of AIS providers at large beyond these institutions. Despite this, our 60% threshold suggests with 99% confidence that the results of a larger survey would show over 50% agreement in favor of all the minimum data elements presented in our study. Finally, the recommendations proposed by this study are based on the popular belief of survey respondents and are limited to the clinical evaluation of patients with AIS. Clinical care for patients with other etiologies of scoliosis, such as congenital or neuromuscular scoliosis and other spinal deformities, may require different criteria for adequate evaluation and standardization of data collection.

## Conclusions

This study establishes a foundational framework on critical data elements within clinical AIS visits. Despite the large variability in clinical practices, a minimum data set can be established to answer critical research questions through large multicenter data efforts. The findings from this study serve as the basis for future registry-based studies to improve nonoperative protocols for the study and treatment of adolescent idiopathic scoliosis.
